# Psychiatry and mental health teaching programs of eight portuguese-speaking schools of medicine: A comparative analysis

**DOI:** 10.3389/fpubh.2022.936177

**Published:** 2022-11-07

**Authors:** Maria Rosel Pedro, Antonio Pacheco Palha, Maria Amelia Ferreira

**Affiliations:** ^1^Departamento de Medicina, Faculdade de Medicina, Universidade Eduardo Mondlane, Maputo, Mozambique; ^2^Departamento de Psiquiatria, Faculdade de Medicina, Universidade do Porto, Porto, Portugal; ^3^Departamento de Ciências da Saúde Publica e Forenses e Educacao Medica, Faculdade de Medicina, Universidade do Porto, Porto, Portugal

**Keywords:** psychiatry, mental health, medical education, teaching and assessment methods, program comparison

## Abstract

**Background:**

Improvement of teaching methods in psychiatry has been the subject of permanent adaptation and innovation. Strengthening graduate education skills in psychiatry and mental health will allow physicians to have the knowledge, skills, and attitudes to carry out early diagnosis and treatment at primary healthcare settings, taking into consideration that the population should benefit from the best interventions by general practitioners.

**Objective:**

The objective of this study was to examine how the undergraduate program of psychiatry and mental health subject in the schools of medicine of the Community of Portuguese-Speaking Countries in the three continents is structured.

**Methods:**

The methods include a narrative description of the program of psychiatry, the workload, the delivery and assessment methods, and the ethical and socio-cultural aspects in psychiatry and research made by the director of the course of psychiatry in Portugal, Brazil, and Mozambique.

**Results:**

Eight schools of medicine from Portugal, Brazil, and Mozambique participated in the study. All these schools use standards which are defined by the regulatory bodies of their countries. The teaching year varied between the third and the sixth. The workload varied between 140 and 224 h. Topics were addressed in presence or virtual methods. Combined qualitative and quantitative assessment is done to encompass competencies, skills and knowledge based on clinical histories, ongoing assessment, seminars, and final written tests. Ethical and socio-cultural aspects in various strands are taught to be linked to the local reality. Research is encouraged by using grants.

**Conclusion:**

Teaching psychiatry follows global and national standards and is organized according to the reality of each country. Psychiatry departments from these three continents invest in teaching methodologies that encourage self-knowledge and the development of critical thinking, which is evaluated in a holistic context. The authors consider that the programs should have a workload according to the current burden of mental illness.

## Introduction

The teaching of psychiatry around the world has been taking place in medical schools for over a century and has been improving along time. In addition to improving the content taught, the workload has also been increasing that the students must learn the importance of psychological factors in the social and clinical context, diagnose psychiatric diseases, and be able to provide, at least, primary care for serious conditions such as anxiety, depression, psychosis, epilepsy, and mental retardation. In Africa, the challenge of specialized human resources and dependence on foreign experts persists (1).

Psychiatry Training for Medical Students varies widely across the world. High-income countries mainly implement competency-based medical education (CBME) and incorporate training in psychiatry during the basic/pre-internship period itself. There is more confidence in skills development and flexible learning-based training than time-based training (2).

Regarding the Portuguese-speaking countries, advances in the medical field are also associated with the independence and the development of the countries in which Africans now have more recent universities with < 65 years of general teaching in psychiatry. Portugal has taught medicine for more than five centuries leading to the evolution of psychiatry, whose teaching only began at the University of Coimbra in 1910, although the University itself was created in 1,308. From 1911 onward, there was a more solid teaching of psychiatry in the new faculties of medicine as reflected in the advances achieved in the teaching methods and provision of psychiatric care and primary healthcare (3). The medical courses in Portugal adopted the model of knowledge and skills as defined in the profile of the Graduate Doctor in Portugal (2005), considered essential for the further development of students and for doctors for their patients and the society (4).

Psychiatric education in Brazil began during the asylum era in the second-half of the 19th century and evolved to occupy the university hospital in the 20th century. Medical residency in psychiatry has shown considerable growth since its implementation 70 years ago and now occupies a prominent place in Brazilian medicine (5).

The university model in Brazil, a mirror of the European model, was officially reformulated in 1968 with the approval of the University Reform Law which extinguished the chairs and replaced them with the departmental structure. In the case of the medical course, the obligatoriness of a basic core, common to all courses in the area of health and the professional cycle (6), was determined.

The pre-defined profile objectives of graduates in medicine in Mozambique recommend that the graduates should be capable in terms of knowledge and have skills and abilities in hybrid teaching models of modules and subjects, the first part being theoretical and the second clinical, which includes internships in hospital units and family follow-up (7).

When we look at the mental health component, it is clear that it continues to be a neglected part of the efforts to improve the global health system, where in low- and middle-income countries mental health budgets are <3% of an already scarce health sector provision (8). The objective of improving these teaching methods is, ultimately, to ensure physicians are capable of recognizing and treating the most frequent pathologies that are often not recognized and, consequently, not treated including mental health disorders.

Mental illness has moved from being the thirteenth cause of care in 1990 to becoming the seventh cause in 2019. Neuropsychiatric disorders attribute for over 10% of the global burden of disease, while mental, neurological, and substance use (MNS) disorders are the leading contributors to years lived with handicap (9). While the physical, social, and economic burden of mental illness is immense, the World Health Organization's Mental Health Atlas (2019) reports that the average number of mental health workers globally is nine per 100,000 people with an extreme variation between the low-income and the high-income countries (10).

In Africa, mental illness continues to be evolved in a mystical world in which the supernatural and witchcraft overlap with scientific concepts, despite educational campaigns promoted by government at the community level (11).

For the treatment of these diseases, while all medical schools are obliged to cover the same required outcomes during the course, they differ in the ways they choose how to deliver the contents of psychiatry and mental health (12, 13). The teaching of psychiatry can start in the 1st years of the medical course, be part of transversal modules, or be taught in the last years of the course.

Clinical experience in general adult psychiatry and sub-specialties is provided by each medical school during that period (14). For example, the University of Alberta MD Undergraduate Program provides for a progressive environment where education, research, and patient care are integrated into a unified curriculum to make sure that they can provide students with not only the technical skills and scientific knowledge which will allow them to become competent doctors but also to be kind and empathetic physicians (15).

To improve knowledge of psychiatry, the RCPsych has recently encouraged medical schools “to put mental health at the heart of the curriculum” (RCPsych 2017). This suggests amendments to student curricula to offer psychiatry experience as early as possible and in an integrated manner with other specialties (16) since psychiatric disorders are among the world's leading health problems due to their high prevalence and chronic course (17).

### Purpose of the study

The purpose of the study was to examine the undergraduate programs of the subject of psychiatry and mental health in the schools of medicine of the Community of Portuguese-Speaking Countries (CPLP) in three continents regarding the standards, delivery and assessment of contents, workload, and teaching methods. Additional information regarding the resources, ethics, and socio-cultural influences in mental health teaching was also analyzed. Major objective is to obtain relevant information for the elaboration of a teaching and evaluation program in psychiatry and mental health that can be used to create and adapt the programs to global needs.

## Materials and methods

### Survey design

A questionnaire was designed on google forms for online completion to expedite data collection.

The survey, consisting of 48 questions, was divided into nine groups (Table 1) taking into account the purpose of the study raised by the researchers and using Kern's Six Step Approach to Curriculum Development for Medical Education, namely (1) Problem Identification and General Needs Assessment, (2) Targeted Needs Assessment, (3) Goals and Objectives, (4) Educational Strategies, (5) Implementation, and (6) Concepts for Evaluating the Effectiveness of the Curriculum.

**Table 1:** Process control and commitment to results.

	**Portugal 1**	**Portugal 2**	**Portugal 3**	**Portugal 4**	**Portugal 5**	**Brazil**	**Mozambique 1**	**Mozambique 2**
Entity that does Accreditation and certification of schools of Medicine	A3ES	A3ES	A3ES	The Ministry of Health and Education evaluate each course and faculty regularly to keep up with medical education standards.	The Ministry of Science and Higher Education. Also the Medical Council	Inserted in the higher education assessment system	Ministry of Higher Education, Science and Technology	Ministry of Higher Education, Science and Technology
Standards used	A3ES standards	A3ES standards	Criteria common to all Universities	WFME standards	Portuguese Society of Medical Education	WFME standards	CNAQ Standards	CNAQ Standards
Frequency of subject evaluation/audit by students	Annually	Each semester	Each semester	Each semester	The school of medicine has a Pedagogical Council in which students participate. It is only when there are educational reforms that students are heard	Each semester	Annually	Annually
Use of audit report made by students	Published on the Faculty website for public domain	Disclosed internally	Global data is published by the Rectory	Published on the Faculty website for public domain	The Pedagogical Council presents the results to the other bodies of the faculty.	Published on the Faculty website for public domain	Used by the Department to improve quality of teaching	Used by the Department
Frequency of subject evaluation by professors	Annually	Each semester	Each semester	Annually	Annually	Each semester	Annually	Annually
Use of the evaluation report done by professors	Improvement of quality contents	Improvement of quality contents	Improvement of quality contents	Improvement of quality contents	Improvement of quality contents	Improvement of quality contents	Improvement of quality contents	Improvement of quality contents
Entity that performed the last external audit and year.	The University. Every five years. 2017. National entity	A3ES. 2019. National entity	A3ES 2018. National entity	A3ES 2019. National entity	A3ES 2019. National entity	The University. 2019. National entity	Office for Academic Reform and Regional Integration (GRAIR) 2015. National entity	The Faculty. 2019. National entity
Means to deliver the audit report	Faculty guardianship	A3ES report	A3ES report	On the Ministries website and in circulation newspapers	A3ES report	Published on the Faculty website for public domain	University council	University council

### Participants

#### Sampling method

The aforementioned study has been carried out upon the invitation of the director of the discipline of psychiatry and mental health of the undergraduate course in medicine, to answer anonymously to an online survey.

More than 20 emails were sent to the departments of psychiatry and mental health in five Portuguese-speaking countries.

The invitation to participate was sent on January 2021. The survey remained open until June 2021, and the date of completion was recorded with each respondent's data.

Eight departments of psychiatry and mental health from Portugal, Brazil, and Mozambique were enrolled.

This online method was selected given the ease of contacting the psychiatry departments of the medical schools. Each survey was accompanied by an online informed consent, which stated the purpose of the study, a confidentiality clause, and the main researcher's contact details.

The selection criteria for the participants included (a) Portuguese-Speaking Country Schools of Medicine, (b) having a Department of Psychiatry and Mental Health, and (c) agreeing with the referred consent statement.

#### Type of the study

A qualitative cross-sectional study, using a narrative description of the delivery and assessment of the Psychiatry and Mental Health Program used by the CPLP schools of medicine.

The answers to the online questionnaires were organized in a spreadsheet with the data.

## Results

Although there is no ideal model that can be applied in all countries, the implementation of specific standards and core curriculums in medical education as well as academic development programs for the teaching staff are important elements for a proper definition of the discipline.

All departments of psychiatry and mental health participating in the current study have an entity that carries out certification and accreditation. It can, therefore, be said that accreditation is a process that is of great interest to all educational institutions as it allows (1) to determine whether an institution meets minimum quality standards; (2) allows students to determine which institutions are credible for enrollment; (3) assists institutions in determining the essential credits for transfer; (4) helps employers determine the validity of study programs and whether a graduate is qualified; and (5) gives employers evidence that applicants have received a degree from an accredited school or program (18).

The institutions which have been surveyed regularly carried out self-assessment audits and external audit processes, discussing at the level of the pedagogical management, publishing on the university websites or in newspapers the results of these assessments.

### Department of psychiatry organization

About 87.5% of the schools of medicine teach the discipline of psychiatry in the 5th year, after the pre-clinical years, and after the subject of Internal Medicine and Psychology, guaranteeing a basic knowledge on human behavior, of the determining factors in the emergence of mental illnesses and the relevant psychological information leading to psych diagnosis and psychological interventions.

**Figure 1:**
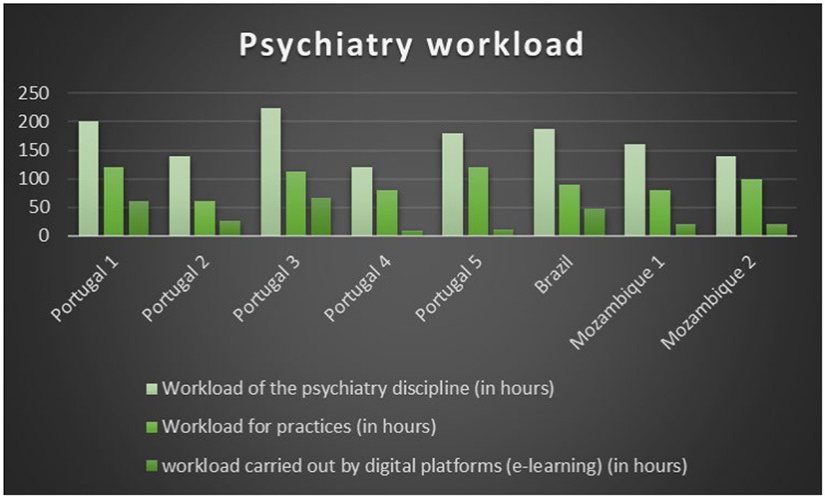
Workload of the psychiatry subject.

The responsibility for preparing the classes is shared by the professors of the Department of Psychiatry, the workload varying from 120 h (Portugal 1) to 224 h (Portugal 3), divided between theoretical, theoretical–practical, and practical classes. In terms of delivery services, despite the restrictions imposed by the COVID-19 pandemic that led to confinement and isolation and forced a change and reinvention in teaching methods, only one of the departments uses half of its psychiatry time for classes by remote means of teaching (Portugal 1). Differences also lie in the time dedicated to practical classes, which vary between 60 and 120 h (Table 2, Figure 1).

**Table 2:** Organization of the psychiatry subjects.

	**Portugal 1**	**Portugal 2**	**Portugal 3**	**Portugal 4**	**Portugal 5**	**Brazil**	**Mozambique 1**	**Mozambique 2**
Year in which Psychiatry is Taught	Fifth and Sixth Years	Fifth and Sixth Years	Fifth Year	Fourth Year	Fourth and Sixth Years	From Third to Sixth Years	Fifth Year	Fifth Year
Mandatory Subjects that precede Psychiatry	Psychology	Pre-Clinical Years are Mandatory	Psychology & Introduction to Clinical Practice	None	Propaedeutics has a Medical Psychology Module before Psychiatry	Internal Medicine	Internal Medicine	Internal Medicine
Monitoring of Classes and Internships	Psychiatric Assistants from the Department	Visiting Professors	Psychiatric Assistants from the Department	Psychiatric Assistants from the Department	Psychiatric Assistants from the Department	Psychiatric Assistants from the Department	Psychiatric Assistants from the Department	Psychiatric Assistants from the Department
Teaching Delivery Methods	Classroom classes; Group and Individual Classes, Online Group and General Classes Internship	Classroom Classes; Group and Individual Classes; Online Group and General Classes; Internship	Classroom Classes; Group and Individual Classes; Online Group and General Classes; Internship	Classroom Classes; Group and Individual Classes Online Group and General Classes; Internship	Classroom Classes; Group and Individual Classes; Online Group and General Classes; Internship	Classroom Classes; Group and Individual Classes; Online Group and General Classes; Internship	Classroom Classes; Group and Individual Classes; Online Group and General Classes; Internship	Classroom Classes; Group and Individual Classes; Online Group and General Classes; Internship
Planning of Classes	A Group defined for that Purpose	Department of Psychiatry and Mental Health and other Specialties, in an Integrated Manner	A Group defined for that Purpose	Department of Psychiatry and Mental Health and other Specialties, in an Integrated Manner	Department of Psychiatry and Mental Health and other Specialties, in an Integrated Manner	Department of Psychiatry and Mental Health and other Specialties, in an Integrated Manner	Department of Psychiatry and Mental Health and other Specialties, in an Integrated Manner	Department of Psychiatry and Mental Health and other specialties, in an Integrated Manner
Workload of the Psychiatry Subject	200 h	140 h	224 h	120 h	180 h	188 h	160 h	140 h
Workload for Practices	120 h (60%)	60 h (42%)	112 h (50%)	80 h (66.6%)	120 (66.6%)	90 h (47%)	80 h (50%)	100 h (71%)
Workload carried out by Digital Platforms (e-learning)	60 h	26 h	66 h	10 h	12 h	47 h	40–60 h	20–40 h

### Psychiatry delivery

Most of psychiatry departments have a focus on psychotic disorders (including schizophrenia), affective disorders, and substance abuse disorders with the time allotted to each varying.

As a reference, four psychiatry departments use the WHO International Classification of Diseases version 10 (ICD-10) (50%) (Portugal 1, Portugal 3; Mozambique 1, Mozambique 2). Two departments (25%) use a combination of the International Classification of Diseases version 10 and the Diagnostic and Statistical Manual of Mental Disorders, Fifth Edition (DSM V) (Portugal 2 and Brazil). One department (12.5%) uses the International Classification of Diseases version 11 (ICD 11) (Portugal 5). One department (12.5%) uses the American Classification of Mental Illness DSM V (Portugal 4) as presented in Table 3.

**Table 3:** Delivery of psychiatry teaching.

	**Portugal 1**	**Portugal 2**	**Portugal 3**	**Portugal 4**	**Portugal 5**	**Brazil**	**Mozambique 1**	**Mozambique 2**
Syllabus of the Psychiatry and Mental Health discipline	Major Psychopathological Syndromes	Main Psychiatric nosography	(Includes adults and child psychiatry): Clinical history and psychopathology + main nosologies + therapeutic approach	Psychopathology, clinical psychiatry and therapeutic methods	Psychiatric syndromes, clinical history, therapeutics in psychiatry	Psychological development throughout the life cycle, main psychiatric pathologies	Psychiatric nosography, clinical history, therapeutics in psychiatry	Major psychiatric syndromes.
Reference Classification of Mental Illness used	CID 10	CID10 and DSM V	CID 10	DSM V	CID 11 and DSM V	CID10 and DSM V	CID 10	CID 10
Specific topics of ethics and deontology in Psychiatry and Mental Health addressed	Ethics and Mental Illness, treatment and doctor-patient relationship	Patient autonomy, confidentiality, doctor-patient relationship, compulsive treatment	Consent, Mental Health Law, Compulsory internment, medical professionalism	Ethics in care, bioethics and ethics in research.	No specific topics	Ethics in psychiatry	No specific topics	No specific topics
Sociocultural aspects of the region and/or country with the most impact on the path of the population of your region	Employment, family relationships, rurality	Religion, family tradition, rural tradition	Socio-economic level, Health care system, Level of education	Violence, socio - economic and religion	Substance dependence (alcoholism, drugs of abuse)	Ethnic minorities, cultural minorities, migrants	Sorcery implicated in the cause of mental illness, Seek medical assistance after permission from the healer, Daughters-in-law need permission from mother-in-law to seek medical care	Mental illness Synonym of spell/evil spirits/punishment, Medical assistance conditioned by the permission of a healer, Contagious disease
Addressing traditional beliefs about mental illness	Yes	Yes	Yes	Yes	In the 3rd year, in the Medical Psychology module.	Yes	Yes	Yes

In terms of ethics topics taught, all departments cover ethics topics with the exception of departments 7 and 8 (Mozambique), namely ethics and mental illness, treatment and doctor–patient relationship, compulsory internment, ethics in care, bioethics, and ethics in research.

In the Table below, we can see that the socio-economic contents are also addressed, taking into account those that are most pressing in the countries enrolled:

### Research in psychiatry and mental health

All departments carry out research in psychiatry, and the present work maintains that the research activities in psychiatry are a crucial factor for the development and updating of the teaching program and the improvement of the quality of training that brings about changes in the quality of professionals committed to the mental healthcare of the population, in accordance with the demands of contemporary society, respecting diversity and cultivating solidarity, inclusion, human values, and ethics and contributing to the formation of qualified citizens who, in turn, will promote sustainable development (14, 19).

### Student performance evaluation

Departments use blended teaching methods to assess students' knowledge, skills, and attitudes that include clinical histories, class participation, and tests throughout the class period and at the end of the discipline/module (Table 5).

## Discussion

### Importance of the subject of psychiatry for the medicine degree

The teaching of psychiatry in schools of medicine has been evolving, and the last decades have brought great changes in the implemented delivery and assessment methods (4). Due to the increasing burden of mental illness globally, recognition of this specialty allows for better healthcare (9).

Students' experience of psychiatry at medical school will influence not only their career choices but also their lifelong attitudes toward people with mental health problems, whatever their specialty (20, 21). Medical education in psychiatry paves the way to gains from educational innovations although it has been slow on the uptake (22).

### The control of quality of schools of medicine

The existence of entities to control higher education is one of the mechanisms to support the quality of higher education and the commitment of the schools to deliver the best education possible.

In the three countries mentioned below, the schools of medicine and the higher education system have regulatory bodies.

In Portugal, the Agency for the Assessment and Accreditation of Higher Education (A3ES) (23), through its regulatory framework, supervises higher education activities, with the agency having the decision-making independence in relation to certain procedures related with the curricula in force, while in Brazil, this responsibility belongs to the National Higher Education Assessment System (SINAES)^25^. In Mozambique, the National Council for the Assessment of Higher Education Quality CNAQ) accredits and certifies the institutions of higher education (24).

The European Association for Quality Assurance in Higher Education (25) and the Faculty of Medicine of the University of Geneva (2006) (26) in the process of reinforcing the quality of teaching promote evaluation mechanisms, namely self-evaluation, in which faculty students and administrative staff make a collective internal reflection on the institution's *status quo* and its practices that provide crucial information to the managers, course directors, and university professors about the institution's state that can lead to appropriate improvements.

### Delivery of psychiatry contents

In the most of the curriculums, psychiatry is taught in the last years of medical school. However, early exposure to this vital specialty can be achieved through the integration of introductory psychology and psychiatry topics during the early college years into other specialties (27).

In the present study, we found that psychiatry and mental health programs in the eight schools of medicine have several common aspects in terms of the program syllabus, practical classes, internship, and the use of objective structured clinical examination.

The programs differ in the number of hours devoted to psychiatry and in the teaching methods used, which shows the importance that is given to psychiatry in different areas of the world.

Logistics and financial conditions for the improvement of teaching quality represent a commitment on the part of the institution to ensure that lecturers and students have adequate means to perform their duties: indicating the way, not giving the answers, accompanying the students according to the objectives defined for the course and the student—to be able to develop skills to develop autonomy, self-learning, and critical thinking.

The study results presented several approaches to deliver psychiatry classes to undergraduate medical students in different settings which included seminars, theoretical classes, practices, and case analysis.

We consider that the success of the psychiatry and mental health program at schools of medicine depends a lot on the defined program content, way of implementation, as well as the selection of adequate and appropriate methods to assess knowledge, skills, and attitudes. Rather than the traditional lectures, it will be more effective to use role play, appropriate use of audio-visual aids, learning by doing, and asking questions to interest the students (21, 28). It is important that students have greater practical training, in a diversity of training scenarios that will allow them to progressively integrate their paths in hospitals to meet curriculum reform “based on health systems” (29).

Dale et al. (30) consider that the teaching of psychiatry should include the introduction of students to psychiatric phenomenology, and the ability to carry out a mental status examination for the diagnosis of major mental disorders, forensic psychiatry, cross-cultural psychiatry, intellectual handicap and mental health, gender aspects in psychiatry, religion, and spirituality in psychiatry (31) indicates that eventually the medical student, the future general practitioners, will not receive any other formal education in psychiatry and, after training in medical practice, will receive users from almost all areas of specialty. Due to the aspects mentioned, it is therefore important to have a basic education that covers a diverse range of pathologies.

Knowing that depression, anxiety, and schizophrenia are among the leading causes of burden worldwide (ranked 13th and 24th leading causes of DALYs, respectively) and the ongoing impact of the COVID-19 pandemic, it is likely that the global burden of mental disorders will increase (9, 32). Effective responses to this burden are achieved by reinforcing mental health interventions at all levels, including undergraduate teaching, to ensure that medical doctors that work at primary levels of healthcare facilities are capable of intervening by recognizing and treating these patients (33, 34).

Considerable discrepancies in content coverage, the delivery of ethics, and socio-cultural themes in psychiatry and in assessment methods were also found.

In this chapter, we make use of Culver et al. (35) who propose that the program of the discipline should not be limited only to the ethical aspects in psychiatry but must also include the religiosity of communities for a better understanding and perception of mental disorders, and on this matter, culture is increasingly becoming an issue for mental healthcare provider (36). Local practitioners and policymakers actively neglect cultural determinants in the mental health field (37). The imaginary of ethnic uniformity is a barrier to the implementation of culturally sensitive interventions (37). In Africa, the influence of traditional concepts is great. If certain conceptions and practices have proved their effectiveness in treating mental disorders, others, on the contrary, participate in promoting the emergence of mental health diseases. In this region, it is believed that witchcraft, sorcery, spells, and spirits can lead to mental illness (38, 39). Attention to psychosocial and socio-cultural aspects in mental health seeks to point out new paths in physical health and mental health that challenges training institutions, health managers, and research (40). Paying attention to these aspects allows for a better understanding of the *ego* of the individual who presents himself to physicians searching for assistance and care (17, 41, 42).

To classify the mental health disorders, there are two major diagnostic manuals: the International Classification of Diseases 10 and the Diagnostic and Statistical Manual of Mental Disorders, which provide classification systems which are relevant to public health, clinical diagnosis, service provision, and specific research applications (43, 44). The International Classification of Diseases 10 alone is the most used (50%), and in combination with the DSM V, it corresponds to 75% of the classification used in these schools of medicine. This is followed by DSM V, which corresponds to 50%, but in combination with ICD 10 or ICD 11.

### Can *e-learning* be improved?

The challenge of using Information and Communication Technologies is pressing, and the pandemic caused by the SARS-CoV-2 virus has forced institutions to adopt hybrid teaching styles, favoring virtual laboratories, among others, and psychiatry is no exception. Undergraduate training programs in psychiatry can benefit if they are adapted to this new reality that requires the use of virtual learning and recommends that the schools enhance different aspects of the educational process that improves interactivity while using the virtual platform in teaching (45). It is worth mentioning creation of mechanisms that ensure that students have resources (computers, mobile phones, and Internet network) to access classes (46, 47).

### Research in undergraduate psychiatry discipline

Loema (48) states that changes in psychiatry can only occur when they are based on scientifically proven assumptions that allow changes to be made for health promotion, prevention, and treatment of diseases, this being the pillar of medicine.

There is need to improve research in psychiatry and support its implementation to provide platforms for our better understanding and improvement of learning, teaching, and assessment methods that can be achieved with reforms that can increase financial support and resources to improveinfrastructures and recruit qualified staff that can motivate students (49) (Table 4).

**Table 4:** Research at the department of psychiatry.

	**Portugal 1**	**Portugal 2**	**Portugal 3**	**Portugal 4**	**Portugal 5**	**Brazil**	**Mozambique 1**	**Mozambique 2**
Research on a Regular Basis	Yes	It is Encouraged	Depends on External Funding	Yes	Yes	Yes	It is Encouraged	Depends on External Funding
Research undertaken by Students	Yes	No	No	Yes	Yes	Yes	Yes	Yes

**Table 5:** Assessment.

	**Portugal 1**	**Portugal 2**	**Portugal 3**	**Portugal 4**	**Portugal 5**	**Brazil**	**Mozambique 1**	**Mozambique 2**
Assessment Methods, including the Mechanisms for Implementing each one, made known at the Beginning of the Classes	Yes	Yes	Yes	No	Yes	Yes	Yes	Yes
Assessment Based on the Clinical and Epidemiological Dimension of the Country, using Topics of Interest to Clinical Practice, or based on General Theoretical Topics	It is Fundamental the Knowledge of the Incidence of Mental Pathology	More Relevance is given to Prevalent Pathologies such as Dementia and Alcohol Use Disorders	It focuses on the Most Epidemiologically Relevant Nosologies	The Inter-relationship between Mental Disorders and the Different Clinics	There is Practical and Theoretical Assessment based on the Topics Delivered	Considered Clinical and Epidemiological Factors in the Country	Assessments are Carried out on the Basis of General Theoretical Topics	Assessments Carried out based on Theoretical Topics and Practical Classes
Calculation of Final Internship Grade	Written Test	Practical Assessment & Multiple-Choice Test & Final Clinical Skills Test	Continuous Assessment (20%) & Oral Exam (80%)	Through Written (40%) & Oral Test (60%).	Oral Test	Sum of Participation (30%), Report (30%) and Theoretical-Practical test (40%)	Task Grid (60%) & Continuous Assessment (40%)	Sum of Clinical History (40%) and Continuous Assessment (60%)
Assessment of students' skills	Clinical History, Use of OSCE	Practical Assessment & Multiple, Choice Test & Final Clinical Skills Test (OSCE)	Continuous Assessment in the Practical Internship, OSCE	Through Written and Oral Test	In Practical Classes, OSCE	Sum of Participation, Preparation of Report and Theoretical-Practical test, OSCE	Through Evidence in Terms of Knowledge, Skills and Attitudes	The Assessment of Students Skills is based on Evidence through a List of Previously Agreed Tasks with an Emphasis on Knowing how to do, Knowing how to be and being
Assignment of Final Grade of Psychiatry	Average Results of the Theoretical and Practical Evaluations	Average Results of the Theoretical and Practical Evaluations	Average Results of the Theoretical and Practical Evaluations	Average Results of the Theoretical and Practical Evaluations	Average Results of the Theoretical and Practical Evaluations	Average Results of the Theoretical and Practical Evaluations	Average Results of the Theoretical and Practical Evaluations	Average results of Average Results of the Theoretical and Practical Evaluations

### Assessment of students

Last, but not least, the use of reliable and valid assessment methods IS prerequisites for allocating resources to faculty, based on the results of assessments (50, 51).

The formative and summative assessment methods must be in line with the program and teaching methodologies and allow testing of students' knowledge, skills, and competences, taking into account the objectives of each process and theme (52–54).

The use of methods such as the objective structured clinical examination and its adaptations has served as a basis for a critical assessment of the students' knowledge, skills, and attitudes, and its application has been increasing.

The School of Medicine of the University of Lisbon uses an annual and innovative method of assessment. This progress testing assesses students from the same curricular program but in different years. They are submitted to the same test that allows student self-assessment and gives the faculty of medicine information about the stage of knowledge of student positioning within their year as well as their placement in relation to the total number of students who took the examination (55).

Instrumentalization of evaluation makes it possible for the entire teaching–learning process to be also evaluated (56, 57).

### Strengths and limitations and recommendations

The strength of the study was the possibility to explore the differences in three continents with the same language. In addition, the results may express the emphasis on psychiatry that is given by each school of medicine and provide the road maps on how to assure assistance in mental health.

Although we did not find a transcontinental study that can be comparable to this one, we consider that a bigger sample would bring more information to consolidate the findings.

Nevertheless, it is a starting point to promote the study of psychiatry programs and to find common points and differences that can lead to further studies in areas not addressed in the present study.

In the interest of developing a questionnaire that was brief but useful, a number of areas of interest were not explored, which included the topics and undergraduates chosen each year and how the departments prepare the classes and choose the assessment methods and topics as well as how to ensure that the same topic is approached in the same way by the different lecturers during internships and practicals. The impact of migration, war, and natural disasters and the stigma toward mental disorders were not addressed (58). These areas would be important and useful to investigate in the development of this work.

The limitations that we mention above can be used for further research and, above all, to improve the quality of medical assistance.

## Conclusion

The undergraduate teaching of psychiatry as an independent discipline has been gaining more expression, with the reforms taking place in medical schools around the world.

Ensuring that these reforms continue will allow physicians to achieve the greatest possible degree of efficiency in their prophylactic and therapeutic roles and to cover a wider range of psychiatric conditions that are underdiagnosed. The fact that the discipline of psychiatry has a structure, albeit different in each undergraduate medical institution in Europe, South America, and Africa, allows us to continue working in the implementation of methods that improve the acquisition of knowledge, skills, and attitudes and toward the standardization of programs to the standards defined by international and national regulatory bodies. Many challenges in psychiatric education lie ahead.

## Data availability statement

The original contributions presented in the study are included in the article/Supplementary material, further inquiries can be directed to the corresponding author/s.

## Author contributions

MP was involved in all aspects of the research project, design, conducting the research, data handling, exploratory analysis, and drafting and editing of the study. AP and MF assisted with the design of the study and in data interpretation and editing of the study. All authors contributed to the article and approved the submitted version.

## Conflict of interest

The authors declare that the research was conducted in the absence of any commercial or financial relationships that could be construed as a potential conflict of interest.

## Publisher's note

All claims expressed in this article are solely those of the authors and do not necessarily represent those of their affiliated organizations, or those of the publisher, the editors and the reviewers. Any product that may be evaluated in this article, or claim that may be made by its manufacturer, is not guaranteed or endorsed by the publisher.

## Abbreviations

A3ES: Agency for the Assessment and Accreditation of Higher Education
CBME: Competency-based Medical Education
CNAQ: National Council for the Assessment of Higher Education Quality
CPLP: Community of Portuguese Language-Speaking countries
DALYs: Handicap-Adjusted Life Year
DSM V: The Diagnostic and Statistical Manual of Mental Disorders, Fifth Edition
ENQA: The European Association for Quality Assurance in Higher Education
GBD: Global Burden of Disease
ICD 10: International Classification of Diseases - 10
ICD 11: International Classification of Diseases
MNS: Mental, Neurological and Substance Abuse
OSCE: Objective Structured Clinical Examination
SINAES: National Higher Education Assessment System
WHO: World Health Organization
WFME: World Federation for Medical Education
WPA: World Psychiatric Association.
